# Age-specific percentile-based prostate-specific antigen cutoff values predict the risk of prostate cancer: A single hospital observation

**DOI:** 10.37796/2211-8039.1415

**Published:** 2023-09-01

**Authors:** Teng-Fu Hsieh, Hung-Lin Chen, Ying-Fang Hsia, Che-Chen Lin, Hsiu-Yin Chiang, Min-Yen Wu, Sheng-Hsuan Chen, Po-Fan Hsieh, Hsi-Chin Wu, Han Chang, Chin-Chi Kuo

**Affiliations:** a Division of Urology, Department of Surgery, Taichung Tzu Chi Hospital, Taichung, Taiwan; b Big Data Center, China Medical University Hospital, Taichung, Taiwan; c Department of Urology, China Medical University Hospital, Taichung, Taiwan; d Graduate Institute of Biomedical Sciences, College of Medicine, China Medical University, Taichung, Taiwan; e Department of Urology, China Medical University Beigang Hospital, Beigang, Yunlin, Taiwan; f Department of Pathology, China Medical University Hospital, Taichung, Taiwan; g College of Medicine, China Medical University, Taichung, Taiwan; h Department of Medical Research, China Medical University Hospital, Taichung, Taiwan; I Division of Nephrology, Department of Internal Medicine, China Medical University Hospital, Taichung, Taiwan

**Keywords:** Age-specific, Cutoff, Prostate cancer, Prostate-specific antigen, Screening

## Abstract

**Background:**

Testing for prostate-specific antigen (PSA) is often recommended for men with a potential risk of prostate cancer (PCa) before requiring advanced examination. However, the best PSA cutoff value remains controversial.

**Object:**

We compared the predictive performance of age-specific percentile-based PSA thresholds with a conventional cutoff of >4 ng/mL for the risk of PCa.

**Methods:**

We included men who received PSA measurements between 2003 and 2017 in a medical center in Taiwan. Logistic regression modeling was used to assess the association between age-specific percentile-based PSA thresholds and PCa risk in age subgroups. We further applied C-statistic and decision curve analysis to compare the predictive performance of age-specific percentile-based PSA with that of a conventional cutoff PSA.

**Results:**

We identified 626 patients with PCa and 40 836 patients without PCa. The slope of PSA in patients >60-year-old was almost 3 times that of those <60-year-old (0.713 vs 0.259). The risk effect sizes of the 75th percentile PSA cutoff (<60-year-old: 2.19; 60–70-year-old: 4.36; >70-year-old: 5.84 ng/mL) were comparable to those observed based on the conventional cutoff in all age groups. However, the discrimination performance of the 75th percentile PSA cutoff was better than that of the conventional cutoff among patients aged <60-year-old (C-statistic, 0.783 vs. 0.729, *p* < 0.05). The 75th percentile cutoffs also correctly identified an additional 2 patients with PCa for every 100 patients with PSA screening at the threshold probability of 20%.

**Conclusions:**

Our data support the use of the 75th percentile PSA cutoff to facilitate individualized risk assessment, particularly for patients aged <60-year-old.

## 1. Introduction

Although evidence is increasing against routine screening for prostate cancer (PCa) at the population level, early prostate-specific antigen (PSA) tests are still the primary examine for men with an elevated PCa risk in order to make a decision of advanced examination, such as digital rectal examination, biopsy, and magnetic resonance imaging [1]. However, despite considerable research on the topic, the diagnostic and prognostic values of PSA remain uncertain. Conventionally, PSA >4 ng/ mL is considered a critical threshold in the recommendations for prostate biopsy [2]. In more recent guidelines, the decision cutoff for PSA levels has been shifted to 3.1 ng/mL [3]. This adjustment was made to account for the recalibration of PSA immunoassays to the WHO International Standard (IS) 96/670, which led to approximately 20–25% lower biomarker values. However, because of the organ-specific nature of PSA, this threshold tends to overestimate PCa risk, resulting in unnecessary biopsies [4,5] and, consequently, an increased risk of complications such as hematuria and rectal bleeding [6]. The sensitivity of PSA screening is also not high enough to complement its moderate specificity: 23.1% of PCas correspond to PSA <4 ng/mL [7].

Many factors affect PSA values, which decreases the accuracy of PSA screening for PCa diagnosis. For example, diet [8], inflammation in prostate [9], benign prostatic hyperplasia [10], urinary tract infection [11], ejaculation [12], exercise bikes [13] and urological intervention all change the PSA values. When using PSA values as an indicator for PCa screening, we must consider those interfering factors with potential PCa diagnosis.

Approaches used to enhance the predictive performance for PCa of PSA include use of PSA derivatives such as age-specific PSA, PSA density, and PSA velocity; various combinations of PSA molecular forms; and kallikrein biomarker–based risk algorithms such as the 4Kscore [14], prostate health index [15], and Stockholm-3 test [16]. However, using age-specific point-of-care PSA, rather than a universal cutoff, to perform PCa risk assessment is the most practical enhancement [17,18]. PSA levels generally increase with age because older men may have enlarged prostates secreting greater amounts of PSA [19]. Oesterling et al. first proposed age-specific reference ranges for PSA based on a community cohort of 471 healthy white American men in 1993 [20]. Later, studies evaluating age-specific references for PSA in different regions involving various African and Asian community-based populations (Table S1) [20–45] indicated that Japanese, Korean, and Chinese men without PCa had lower PSA distributions than those in Caucasian populations [29]. However, in two hospital-based populations from China and Taiwan, the PSA distribution was similar to that in white American men [30,31]. These observations challenge the generalizability of PSA cutoffs derived from general populations rather than from real-world hospital-based data, which may be the main cause of overestimation of PCa risk. To compare the predictive performance between conventional and hospital-based PSA thresholds, we conducted a large retrospective cohort study based on the 15-year Clinical Research Data Repository (CRDR) of China Medical University Hospital (CMUH), which includes more than 2.8 million patients.

## 2. Methods

### 2.1. Study population

Between January 1, 2003, and December 31, 2017, the CMUH-CRDR documented the details of 2 712 350 patients visiting CMUH. Detailed information on the CMUH-CRDR has been published elsewhere [46,47]. The interoperability of the CMUH-CRDR has further expanded access to national population-based health-related databases (eg, the Catastrophic Illness Database), which are systematically maintained by the Health and Welfare Data Science Center of Taiwan’s Ministry of Health and Welfare. All patients enrolled in the CMUH-CRDR were followed up until December 31, 2017, or death—whichever occurred earlier. All methods in this study were performed in accordance with the relevant guidelines/regulations. This study protocol was approved by the Big Data Center of China Medical University Hospital and the Research Ethical Committee/Institutional Review Board of China Medical University Hospital (CMUH105-REC3-068) and the need to obtain informed consent for the present study was waived by the Research Ethical Committee of China Medical University Hospital.

**Fig. 1:**
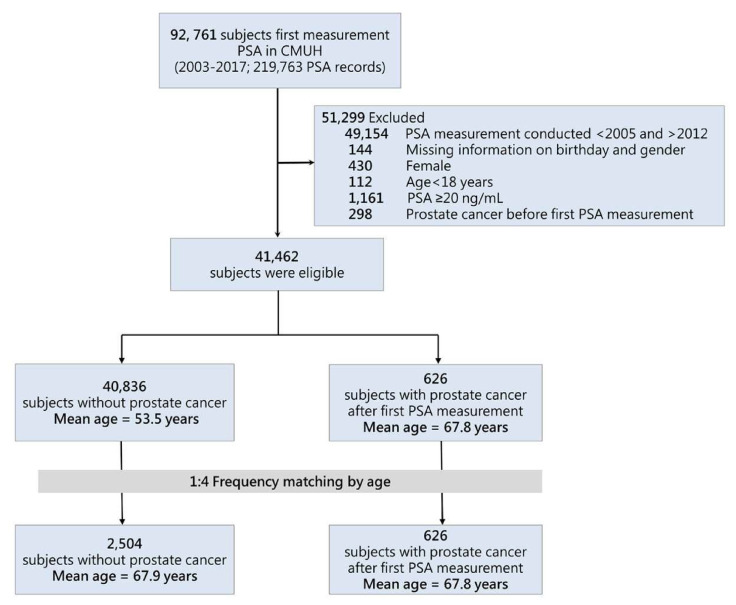
Flow diagram of study population selection. *Abbreviations:* CMUH, China Medical University Hospital; PSA, prostate-specific antigen.

The present study included male patients aged ≥18 years who had PSA measurements in both inpatient and outpatient settings from January 1, 2003, to December 31, 2017 (Fig. 1). We excluded patients who already had a history of PCa at the time of PSA measurement. The index date was defined as the day on which the first PSA was measured at CMUH. To evaluate the association between PSA and PCa by age group, we used frequency matching to match patients with PCa and those without PCa (case:control = 1:4) by age group (overall and <60, 60–70, and >70 years old; Table S2). We also performed a sensitivity analysis on patients who did not have urinary tract infection (UTI) within 6 weeks prior to the index date.

### 2.2. Measurement of PSA

Between 2003 and February 2008, serum total PSA was measured using an automated two-site chemi-luminescent assay with an ultrasensitive PSA reagent kit on the Immulite Third Generation PSA assay system (Diagnostic Products, Los Angeles, CA); after February 2008, it was measured using Hybritech assays on the Access II Immunoassay analyzer (Beckman Coulter, Fullerton, CA). The minimum detectable values before and after February 2008 were 0.003 and 0.008 ng/mL, respectively. In the present study, patients with PSA ≥20 ng/mL were excluded to mitigate the inclusion of patients with potential PCa.

### 2.3. Clinical data captured through CMUH-CDRD and national tracking of PCa

Sociodemographic variables and baseline comorbidities and medications were determined based on the information obtained from the CMUH-CRDR within a 1-year window before the index date. The *International Classification of Diseases (ICD)* codes for comorbidities are listed in Table S3. The use of benign prostatic hyperplasia medications after the first PSA measurement was collected. The PCa result was verified if the patient had a catastrophic illness certificate for PCa. Catastrophic illnesses are defined by Taiwan’s National Health Insurance program, and patients with a catastrophic illness are exempted from copayments. The proportion of missing data is provided in Table S4.

### 2.4. Statistical analysis

Continuous variables are expressed as medians and interquartile ranges (IQRs) and compared using the nonparametric Kruskal–Wallis test or Spearman’s correlation, and categorical variables are expressed as frequencies (percentages) and compared using the chi-square test or Cochran–Armitage trend test. We constructed a scatter plot to visualize the correlation between PSA and age using an age cutoff of 60 years. We also compared PSA distributions by PCa status. The PCa risk was evaluated using univariable logistic regression modeling stratified by age groups of <60, 60–70, and >70 years. To compare the prognostic performance of age-specific PSA for PCa risk, we applied the C-statistic to construct a receiver operating characteristic (ROC) curve and decision curve [48]. The conventional PSA cutoff, 4 ng/mL, served as the reference for the prognostic performance of new cutoffs of the 75th, 90th, 95th, to 99th percentiles of the age-specific PSA distribution. We further characterized the dose–response association with PCa risk by using a restricted cubic spline model with 3 knots located at the 10th, 50th, and 90th percentiles of the overall and age-specific distribution for PSA with reference points set at the conventional and 75th percentile cutoff points. All statistical analyses were performed using SAS version 9.4 (SAS Institute, Cary, NC) and R version 3.5.1 (R Foundation for Statistical Computing, Vienna, Austria). The two-sided significance level of α was set to 0.05.

## 3. Results

Between 2005 and 2012, 40 836 patients without PCa were identified with mean age of 54.3 ± 15.0 years and mean PSA level of 1.8 ± 2.5 ng/mL. A clear increasing trend of PSA with age was observed (*P* for trend <0.001). In >40% of patients, PSA was assessed at the age of 50–70 years (Table 1). Prior to the age of 50, most PSA examinations were performed at the Health and Prevention Center or Division of Family Medicine; by contrast, in patients older than 70 years, PSA testing was generally performed at the urology department or other medical specialty departments (Table 1). Comorbidities such as diabetes, hypertension, and chronic kidney disease were much more prevalent among patients aged >70 years. 5α-reductase inhibitors were rarely used at the time of first PSA testing. The most commonly used medication for possible benign prostatic hyperplasia at baseline was tamsulosin, which was also more frequently prescribed in patients aged >70 years. A clear increasing trend of PSA with increasing age was observed (*P* for trend <0.001). In the scatter plot, the slope for patients older than 60 years was nearly 3 times that of those younger than 60 years (0.713 vs 0.259; Figure S1).

**Fig. 2:**
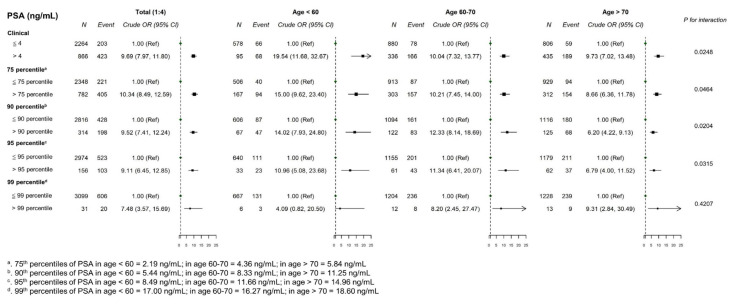
Odds ratio (ORs) for prostate cancer in the 1:4 matched case–control sample based on different prostate-specific antigen (PSA) cutoffs. *Abbreviations:* OR, odds ratio; PSA, prostate-specific antigen.

**Fig. 3:**
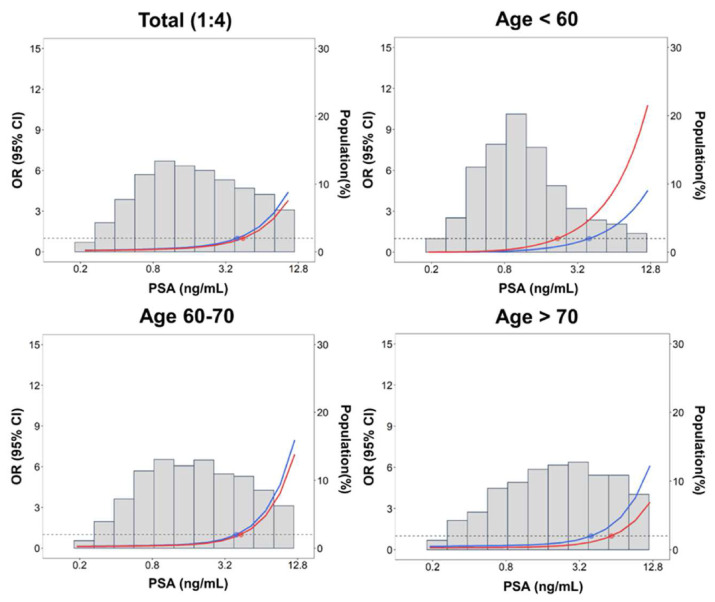
*Odds ratios for prostate cancer mortality by prostate-specific antigen (PSA) levels in the 1:4 matched case*–*control sample*. Solid lines represent odds ratios based on restricted quadratic splines with 95% confidence interval using knots at the 10th, 50th, and 90th percentiles with reference points of 4 ng/mL (blue) and the 75th percentile value (red). Values of the 75th percentile of PSA: 4.44 ng/mL for the overall population, 2.19 ng/mL for age <60 years, 4.35 ng/mL for 60–70 years, and 5.86 ng/mL for >70 years. *Abbreviations:* OR, Odds ratio; PSA, prostate-specific antigen; CI, confidence interval.

**Fig. 4:**
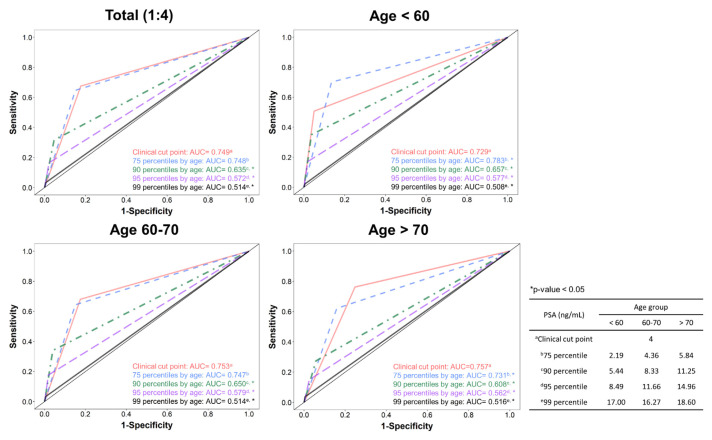
Receiver operating characteristics (ROC) curves of difference prostate-specific antigen (PSA) cutoff values for the risk of prostate cancer in 1:4 matched case–control samples. *Abbreviations:* PSA, prostate-specific antigen; AUC, Area under the Curve.

**Fig. 5:**
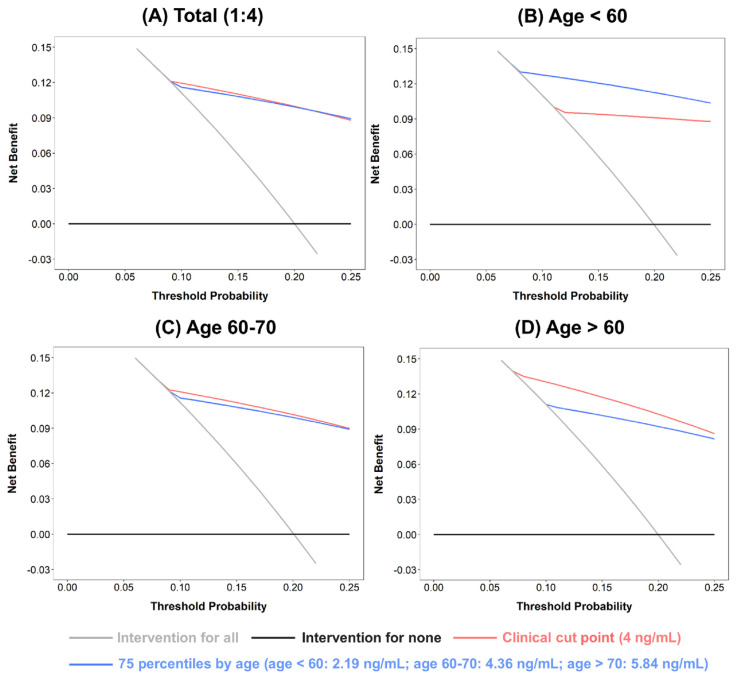
Decision curve analysis for conventional cutoff and the age-specific 75th percentile prostate-specific antigen (PSA) values of prostate cancer.

The comparison of PSA distributions between patients with and without PCa revealed that the PSA level of each selected percentile (50th, 75th, 90th, and 95th) was much higher in patients with PCa. For example, among patients aged 50–59, the median PSA in patients with PCa was >4 ng/mL, whereas the 90th percentile of PSA in patients without PCa was <4 ng/mL (Figure S2). In the 1:4 matched case–control analysis, the conventional PSA cutoff of 4 ng/mL was significantly associated with PCa risk in all 3 age groups (<60, 60–70, and >70 years), with the effect size being close to the odds ratio of 10. In particular, in patients <60 years old, the effect size was markedly high, at 19.5 (95% confidence interval, 11.7–32.7). Using the percentile threshold, the risk patterns of the 75th percentile cutoff were the most similar to those observed based on the conventional cutoff (Fig. 2). The dose–response relationship between PSA and PCa risk displayed a threshold–response pattern (Fig. 3). The predictive performance between the 4-ng/mL cutoff and the 75th percentile cutoff were generally comparable, except among patients aged <60 years in which the area under the ROC curve was significantly greater for the 75th percentile cutoff than for the 4- ng/mL cutoff (0.783 vs. 0.729, *p* < 0.05) (Fig. 4). Patients with UTI affected the PSA levels, usually increased PSA level [49]; accordingly, the UTI effect in sensitivity of PSA levels was assessed. Sensitivity analysis of 2841 patients without UTI within 6 weeks prior to the index date revealed that the PSA distribution was similar to that in the original case–control population, with a median of 1.73 ng/ mL and mean of 3.29 ng/mL (Table S5). In addition, subgroup analysis of UTI status demonstrated that the effect size of PCa risk associated with PSA cutoffs was comparable between the original case–control population and patients without UTI (OR = 10.89, 11.51, 10.30, 9.65, and 9.35 for cutoffs of 4 ng/mL, 75th, 90th, 95th, and 99th percentiles, respectively; Table S6). However, for patients with a history of UTI, the effect size of PCa risk was much lower, especially for PSA cutoffs of 4 ng/mL and the 75th percentile (OR = 3.96 for 4 ng/mL; 4.51 for 75th percentile). In the decision curve analysis, the conventional and 75th percentile cutoffs indicated comparable net benefits (Fig. 5A). With stratification by age group, the 75th percentile cutoff correctly identified an additional 2 patients with PCa for every 100 patients undergoing PSA screening at the threshold probability of 20% compared with the conventional cutoff among patients aged <60 years (Fig. 5B).

## 4. Discussion

Our results suggest the practice of using age-specific PSA cutoffs based on the 75th percentile threshold for patients aged <60 years in the Taiwanese population. The predictive performance of the conventional PSA cutoff of 4 ng/mL and the 75th percentile threshold is comparable among patients aged 60–70 years, but that of the conventional cutoff was better in patients older than 70 years. Our data indicate that using an age-specific percentile PSA threshold improves risk assessment of PCa, particularly among middle-aged men.

The PSA reference range has been defined in previous studies as ≤4 ng/mL in healthy men [50–53]. Huang et al. indicated that the positive predictive value of PSA >4 ng/mL to distinguish organ-confined PCa from benign prostatic hyperplasia was 75% [50]. However, organ-confined PCa can present with total PSA values within the reference range, thereby compromising the sensitivity of PSA testing as a screening tool [54,55]. By contrast, a large proportion of men with PSA levels >4 ng/mL but without PCa was also observed in a systematic review, indicating that the specificity of PSA testing was 0.06–0.66 [56]. However, in real-world settings, PSA remains widely used as the first-line screening test for PCa and, in Taiwan, it is required for the reimbursement of 5α-reductase inhibitors [57,58]. Our data indicated a threshold–response relationship between PSA and PCa risk, implying that the use of a PSA cutoff as the initial screening test is a reasonable practice. The better predictive performance of PCa by using an age-specific cutoff (75th percentile) for patients aged <60 years observed in our study supports the use of age-specific cutoffs for PSA to facilitate individualized risk assessment.

The regional differences in the PSA distribution highlight the importance of establishing regionspecific reference PSA data even among populations of the same ethnicity, particularly in large countries. In China, the 95th percentile of the population PSA was 3.2 ng/dL in Shanghai, 2.92 ng/dL in Beijing, 2.90 ng/dL in Shandong, and 2.35 ng/dL in Shaanxi (Table S3). By contrast, in small countries, such as Taiwan, the difference can be ignored. For instance, in a study from Northern Taiwan, the 95th percentile of PSA in patients aged 50–59 years who underwent routine health checkups was 3.3 ng/dL [31]; in our study involving a Central Taiwanese population, this value was 3.2 ng/dL. The variation in PSA distribution among populations of the same ethnicity may be due to the heterogeneity of methods of enrolling participants, such as differences in clinical settings. However, environmental and dietary factors, such as dietary fat or protein intake, may also affect the population PSA level [59,60]. A recent study of the US general population indicated a nonlinear positive relationship between PSA level (log transformed) and dietary protein intake [60]. However, Hoffmann et al. identified 19 novel single-nucleotide polymorphisms (SNPs) and then integrated them with 21 previously known PCa-related SNPs to form 40 PSA SNPs, which contribute up to 9.5% of the variation in PSA level [61]. Accordingly, future studies should advance the practice of individualized PSA threshold values through the integrated analysis of both genetic and environmental factors.

On the basis of opportunistic single PSA values, we observed that regardless of whether a conventional or percentile threshold is used, the area under the best ROC curve was only approximately 0.75. The Göteborg randomized PSA screening trial indicated that Organized screening reduces PC mortality but opportunistic PSA testing offers little if any survival benefit to PCa and may lead to an over-diagnosis of PCa [62]. However, the European Randomized Study of Screening for Prostate Cancer study group stated that planned PSA screening through structured population-based PSA screening may reduce PCa mortality after a 16-year follow-up [63,64]. To clarify the optimal approach to PSA testing, definitively determine whether regional differences exist, and justify the current risk-adapted strategies and counseling protocol, a global data collection network is warranted. For instance, in the United Kingdom, the latest Prostate Cancer Risk Management Programme recommends that general practitioners refer patients to urologists if a well-informed patient aged ≥50 years has a PSA level >3 ng/mL [65]. Nonetheless, this practice is inconsistent with the current guidelines of the European Association of Urology [1].

This study has several limitations. First, misclassification bias cannot be completely excluded because the diagnosis of PCa relied on diagnostic codes and catastrophic illness certification. Patients with slowly progressing PCa may have been misdiagnosed as not having PCa. However, we extended the follow-up window to ≥5 years, thus minimizing the impact of misclassification. Second, our data used the Siemens and Beckman assays for measuring PSA levels. The Beckman and Siemens assays underestimated PSA levels by approximately 20% and 16% respectively compared to the Roche assay [66]. Method bias can lead to underestimation in initial screening, increasing the risk of missing prostate cancer if risk thresholds are not properly adjusted. Third, we excluded patients with active prostatitis and UTI by using the cutoff of PSA ≥20 ng/mL, but we did not use *ICD* codes at the time of PSA measurement to maintain generalizability, which might have overestimated the PSA threshold. We further performed sensitivity analysis in patients without diagnoses of prostatitis or UTI within 6 weeks prior to the index date, and the overall PSA distribution and the effect size of PCa risk remained similar (data not shown). Third, the single-center study design limits the generalizability of our findings. Future studies should replicate the conceptual framework of the present study in another setting with a special focus on risk–benefit assessment.

The threshold–response relationship observed in the present study supports the practice of using an age-specific cutoff value for PCa screening. For patients younger than 60 years, the age-specific 75th percentile threshold should be used for predicting PCa risk rather than the conventional cutoff of 4 ng/ mL. Our findings may encourage health care institutions to replicate our approach and establish their regional PSA epidemiology, thus improving PCa risk assessment with a single PSA test.

## Appendix

**Table S1:** Mini review of the literature of prostate-specific antigen (PSA) level in different age groups.

Year	Country (Area)	Exclusion criteria/Study design	N	95 percentile PSA level (ng/mL) in age group (year)						Reference

				<40	40–49	50–59	60–69	70–79	>80	
**Region of the Americas (AMR)**										
**1993**	USA (Minnesota) ^a^	PCa/Cross-sectional	471	–	2.5	3.5	4.5	6.5	–	[ 20]
**1996**	USA (White)	PCa/Cross-sectional	1802	–	2.1	3.6	4.3	5.8	–	[ 21]
**1996**	USA (African-American)	PCa/Cross-sectional	1673	–	2.4	6.5	11.3	12.5	–	[ 21]
**African Region (AFR)**										
**2016**	Nigeria ^a^	LUTS and DRE abnormalities/Cross-sectional	4035	–	4.78	5.47	8.93	–	–	[ 22]
**Western Pacific Region (WPR)**										
**1995**	Japan ^a^	Prostate history or current abnormalities/Cross-sectional	286	–	2	3	4	5	–	[ 23]
**1995**	Japan ^a^	PCa/Cross-sectional	2820	–	2.1	2.9	4.0	5.2	5.9	[ 24]
**2000**	Korea	History of PCa or prostate surgery/Prospective study	5805	1.8	2.0	2.5	3.9	6.3	–	[ 25]
**2007**	Korea	History of PCa or prostate surgery/Prospective study	120 439	1.88	1.92	2.37	3.56	5.19	–	[ 26]
**2013**	China (Beijing)	PCa, prostate surgery, urinary tract infection and obstruction/Cross-sectional	1572	–	1.57	2.92	4.11	5.56	7.29	[ 27]
**2011**	China (Shandong)	PCa, prostate surgery, UTI and DRE abnormalities/Cross-sectional	9358	1.89	2.19	2.88	4.42	6.52	–	[ 28]
**2004**	China (Shaanxi) ^a^	PCa, prostate surgery, and prostatitis/Cross-sectional	1096	1.20 1.21	1.23	2.35	3.20	3.39 ^b^	–	[ 29]
**2009**	China (Shanghai)	History of PCa/Cross-sectional	8422	–	2.15	3.20	4.10	5.37	–	[ 30]
**2010**	Taiwan	PCa, UTI, or prostate infection/Cross-sectional	7803	–	2.17	3.33	5.12	6.24	6.61	[ 31]
**2000**	Singapore ^a^	Prostate history or current abnormalities/Cross-sectional	513	1.36	1.73	2.25	4.05	6.30	6.60	[ 32]
**South-East Asian Region (SEAR)**										
**2007**	India (Gujarati) ^a^	Biopsy-proven malignancy/Prospective study	1899	–	2.1	3.4	4.2	5	–	[ 33]
**2004**	South India (South Indian) ^a^	Urological complications/Cross-sectional	583	0.9	1.3	1.48	1.6	2.0	2.47	[ 34]
**2014**	India	Urological complications, post-operation of prostate gland, history of prostate diseases, chemotherapy or radiotherapy, anti-androgen drugs, and having pus cell more than 4 in urine analysis/Cross-sectional	1253	0.71	0.85	1.13	1.45	1.84	2.35	[ 35]
**Eastern Mediterranean Region (EMR)**										
**2018**	Northern Iran	history of prostate cancer, prostate infection and surgery/Cross-sectional	950	0.62	0.75	0.91	1.33	1.45	1.93	[ 36]
**2005**	South Iran ^a^	history of prostate cancer, bladder outlet obstruction, bacterial prostatitis, urinary tract infection, inflammation (eg, pyuria), history of prostate surgery, or recent transurethral procedures/Cross-sectional	650	–	1.3	1.85	3.20	4.4 ^b^	–	[ 37]
**2020**	Iran (Amirkola) ^a^	Aged ≧60 years, cognitive impairment/Cross-sectional	837	–	–	–	4.89 4.88	9.01 7.95	11.98 33.17	[ 38]
**2003**	Saudi Arab	prostate cancer/Cross-sectional	543	–	2.85	3.99	5.41	6.29	6.84	[ 39]
**2019**	Saudi Arab ^a^	history of prostate cancer, prostatitis, urinary tract infection, chronic retention, or coagulopathies/Cross-sectional	7814	1.88	2.12	3.00	3.8	6.9 ^b^		[ 40]
**2003**	Jordan	DRE and TRUS abnormalities, the presence of prostate cancer/Cross-sectional	1852	2.26 2.90	3.15 3.75	3.80 3.76	4.31	–	–	[ 41]
**2012**	Syria ^a^	Aged ≧40 years, history of prostate cancer, urinary tract infection, or prostate surgery/Cross-sectional	2893	–	1.7	2.3	4.8	5.8	–	[ 42]
**European Region (EUR)**										
**2005**	Turkey ^a^	Benign prostate hyperplasia/Cross-sectional	255	–	4.51	4.36	6.17	10.18 ^b^	–	[ 43]
**2010**	Spain ^a^	Aged >64 years and prostate pathology/Cross-sectional	63 926	1.4	1.7	3.3	5.18	–	–	[ 44]
**2012**	Irish ^a^	clinical evidence of PCa by DRE/Cross-sectional	660	1.57 1.65	1.85 2.17	2.63 3.25	4.02 4.96	–	–	[ 45]

Abbreviations: DRE, digital rectal examination; LUTS, lower urinary tract symptoms; PCa, prostate cancer; TRUS, transrectal ultrasound; UTI, urinary tract infection.

a Community-based study.

b Age >70 years.

**Table S2:** Baseline characteristics after 1:4 case–control matching

	Without prostate cancer	With prostate cancer	*P* value ^a^
**Original**			
N	40 836	626	
Age (year), median (IQR)	53.5 (43.5, 64.7)	67.8 (61.4, 73.6)	<0.001
PSA (ng/mL), median (IQR)	0.92 (0.56, 1.73)	6.13 (2.96, 10.30)	<0.001
**Matching**			
N	2504	626	
Age (year), median (IQR)	67.9 (61.3, 73.6)	67.8 (61.4, 73.6)	0.976
PSA (ng/mL), median (IQR)	1.37 (0.72, 2.97)	6.13 (2.96, 10.30)	<0.001
Duration of PSA to prostate cancer			
Mean (Std)	–	2.91 (3.15)	
Median (IQR)	–	1.78 (0.09, 5.32)	
Min-Max	–	0.0–12.8	

Abbreviations: IQR, interquartile range; PSA, prostate-specific antigen; Std, standard deviation.

a *P* values are calculated by Kruskal–Wallis test.

**Table S3:** International Classification of Diseases (ICD) diagnostic codes and medication for comorbidities used in this study

Comorbidity	ICD-9-CM		Medication
**Diabetes mellitus**	250	And	Insulin, Oral antidiabetic drugs (OAD)
**Hypertension**	401–405	And	Angiotensin-converting-enzyme inhibitors (ACEIs), Angiotensin II receptor blockers (ARBs), Diuretics
**Cardiovascular disease**	250.7, 410–414, 425–428, 429.1–429.3, 430–438, 441–442, 443.9, 458, 785.4, V43.4		Not applicable
**Urinary tract infection**	590, 595, 597, 598.0, 599.0, 032.84		Not applicable

**Table S4:** Proportion of missing data in demographic and clinical characteristics by age level in patients without prostate cancer

	Overall	Age<40	40 ≤ Age<50	50 ≤ Age<60	60 ≤ Age<70	70 ≤ Age<80	Age≥80
						
	N = 40 836	N = 2083 (5.1%)	N = 4962 (12.2%)	N = 6587 (16.1%)	N = 10 683 (26.2%)	N = 9312 (22.8%)	N = 7209 (17.6%)
Age (year)	0 (0.00%)	0 (0.00%)	0 (0.00%)	0 (0.00%)	0 (0.00%)	0 (0.00%)	0 (0.00%)
BMI	32 916 (80.61%)	5579 (77.39%)	7570 (81.29%)	8796 (82.34%)	5389 (81.81%)	4014 (80.89%)	1568 (75.28%)
Smoking history	39 276 (96.18%)	7092 (98.38%)	9076 (97.47%)	10 206 (95.53%)	6222 (94.46%)	4729 (95.30%)	1951 (93.66%)
Clinical division	2315 (5.67%)	1135 (15.74%)	694 (7.45%)	435 (4.07%)	46 (0.70%)	5 (0.10%)	0 (0.00%)
Number of PSA measurement	0 (0.00%)	0 (0.00%)	0 (0.00%)	0 (0.00%)	0 (0.00%)	0 (0.00%)	0 (0.00%)
Comorbidity	4758 (11.65%)	2030 (28.16%)	1671 (17.94%)	859 (8.04%)	159 (2.41%)	31 (0.62%)	8 (0.38%)
CKD stage	4879 (11.95%)	257 (3.56%)	703 (7.55%)	1530 (14.32%)	1238 (18.79%)	903 (18.20%)	248 (11.91%)
Baseline medication	12 378 (30.31%)	4001 (55.50%)	4085 (43.87%)	2770 (25.93%)	1017 (15.44%)	399 (8.04%)	106 (5.09%)
Medication after first PSA measurement	4858 (11.90%)	1837 (25.48%)	1713 (18.40%)	939 (8.79%)	266 (4.04%)	75 (1.51%)	28 (1.34%)
**Baseline serum biochemical**							
PSA (ng/mL)	0 (0.00%)	0 (0.00%)	0 (0.00%)	0 (0.00%)	0 (0.00%)	0 (0.00%)	0 (0.00%)
Serum creatinine (mg/dL)	4879 (11.95%)	257 (3.56%)	703 (7.55%)	1530 (14.32%)	1238 (18.79%)	903 (18.20%)	248 (11.91%)
eGFR (mL/min/1.73m2)	4879 (11.95%)	257 (3.56%)	703 (7.55%)	1530 (14.32%)	1238 (18.79%)	903 (18.20%)	248 (11.91%)
ALT (IU/L)	10 144 (24.84%)	357 (4.95%)	1253 (13.46%)	3234 (30.27%)	2567 (38.97%)	2007 (40.45%)	726 (34.85%)
AST (IU/L)	11 482 (28.12%)	403 (5.59%)	1357 (14.57%)	3584 (33.55%)	2902 (44.06%)	2372 (47.80%)	864 (41.48%)
T-CHO (mg/dL)	14 605 (35.77%)	501 (6.95%)	1680 (18.04%)	4237 (39.66%)	3577 (54.30%)	3165 (63.78%)	1445 (69.37%)
TG (mg/dL)	14 603 (35.76%)	501 (6.95%)	1680 (18.04%)	4242 (39.71%)	3573 (54.24%)	3166 (63.80%)	1441 (69.18%)
LDL-C (mg/dL)	17 466 (42.77%)	693 (9.61%)	2211 (23.74%)	5020 (46.99%)	4113 (62.44%)	3735 (75.27%)	1694 (81.33%)
HDL-C (mg/dL)	17 707 (43.36%)	693 (9.61%)	2225 (23.89%)	5067 (47.43%)	4194 (63.67%)	3802 (76.62%)	1726 (82.86%)
**Baseline urine biochemical**							
WBC (uL)	5573 (13.65%)	397 (5.51%)	952 (10.22%)	1526 (14.28%)	1194 (18.13%)	1059 (21.34%)	445 (21.36%)
RBC (uL)	5591 (13.69%)	402 (5.58%)	954 (10.24%)	1531 (14.33%)	1196 (18.16%)	1061 (21.38%)	447 (21.46%)
Epithelial cell (uL)	5591 (13.69%)	399 (5.53%)	954 (10.24%)	1528 (14.30%)	1198 (18.19%)	1067 (21.50%)	445 (21.36%)
Occult blood	5574 (13.65%)	397 (5.51%)	951 (10.21%)	1527 (14.29%)	1194 (18.13%)	1059 (21.34%)	446 (21.41%)
pH	5572 (13.64%)	397 (5.51%)	951 (10.21%)	1526 (14.28%)	1194 (18.13%)	1059 (21.34%)	445 (21.36%)
Crystal (HPF)	5871 (14.38%)	421 (5.84%)	990 (10.63%)	1595 (14.93%)	1252 (19.01%)	1141 (22.99%)	472 (22.66%)
Cast (LPF)	5874 (14.38%)	427 (5.92%)	999 (10.73%)	1590 (14.88%)	1254 (19.04%)	1134 (22.85%)	470 (22.56%)

Abbreviations: ALT, alanine aminotransferase; AST, aspartate aminotransferase; BMI, body mass index; CKD, chronic kidney disease; eGFR, estimated glomerular filtration rate; HDL-C, high-density lipoprotein-cholesterol; HPF, high power field using a microscope; LDL-C, low-density lipoprotein-cholesterol; LPF, high power field using a microscope; PSA, prostate-specific antigen; T-CHO, total cholesterol; TG, triglyceride; RBC, red blood cells; WBC, white blood cells.

**Table S5:** Distribution of prostate-specific antigen (PSA) in patients with and without urinary tract infection (UTI)

Population	N of patients	Minimum	10th percentile	Median (IQR)	90th percentile	Maximum	Mean (SD)	*P* value ^a^
Overall	3130	0.004	0.48	1.79 (0.83, 4.43)	8.97	19.959	3.43 (3.97)	
With UTI Diagnosis within previous 6 weeks before index date	289 (9.2%)	0.008	0.56	2.78 (1.14, 6.83)	12.80	19.7	4.76 (4.87)	<0.0001
Without UTI diagnosis within previous 6 weeks before index date	2841 (90.8%)	0.004	0.47	1.73 (0.81, 4.22)	8.51	19.959	3.29 (3.84)	

Abbreviations: PSA, prostate-specific antigen; UTI, urinary tract infection; IQR, interquartile range; Std, standard deviation.

a *P* values are calculated by Kruskal–Wallis test.

**Table S6:** Subgroup analysis of different prostate-specific antigen (PSA) cutoff values and prostate cancer risk in 1:4 matched case–control sample and stratified by UTI diagnosis history

	Total (1:4)			Without UTI Diagnosis			With UTI Diagnosis			P for interaction
		
	Crude OR			Crude OR			Crude OR			
		
	N	Event		N	Event		N	Event		
PSA (ng/mL) Clinical										
≤4	2264	203	1.00 (Reference)	173	21	1.00 (Reference)	2091	182	1.00 (Reference)	0.0016
>4	866	423	9.69 (7.97, 11.80)	116	41	3.96 (2.18, 7.17)	750	382	10.89 (8.83, 13.41)	
75th										
≤75 percentile	2348	221	1.00 (Reference)	188	23	1.00 (Reference)	2160	198	1.00 (Reference)	0.0035
>75 percentile	782	405	10.34 (8.49, 12.59)	101	39	4.51 (2.50, 8.16)	681	366	11.51 (9.33, 14.20)	
90th										
≤90 percentile	2816	428	1.00 (Reference)	240	36	1.00 (Reference)	2576	392	1.00 Reference)	0.1944
>90 percentile	314	198	9.52 (7.41, 12.24)	49	26	6.41 (3.30, 12.44)	265	172	10.30 (7.83, 13.56)	
95th										
≤95 percentile	2974	523	1.00 (Reference)	256	42	1.00 (Reference)	2718	481	1.00 (Reference)	0.6378
>95 percentile	156	103	9.11 (6.45, 12.85)	33	20	7.84 (3.62, 16.98)	123	83	9.65 (6.53, 14.25)	
99th										
≤99 percentile	3099	606	1.00 (Reference)	284	60	1.00 (Reference)	2815	546	1.00 (Reference)	0.1938
>99 percentile				5	2	2.49 (0.41, 15.24)	26	18	9.35 (4.04, 21.62)	

Abbreviations: PSA, prostate-specific antigen; UTI, urinary tract infection; OR, odds ratio; CI, confidence interval.

a. *P* values are calculated by chi-square test.

Fig. S1 Scatter plot of age at first prostate-specific antigen (PSA) measurement and PSA level among patients without subsequent prostate cancer. *Abbreviations:* PSA, prostate-specific antigen.

Fig. S2 Distribution plot of prostate-specific antigen (PSA) between patients with prostate cancer after first PSA measurement and those without prostate cancer. Shaded area indicates the PSA cutoff of 4 ng/mL for patients aged ≥60 years and 2.19 ng/mL for patients aged <60 years. *Abbreviations:* PSA, prostate-specific antigen.

## Figures and Tables

**Table 1:** Baseline clinicodemographic characteristics stratified by age group among patients without prostate cancer.

	Overall	Age<40	40 ≤ Age<50	50 ≤ Age<60	60 ≤ Age<70	70 ≤ Age<80	Age≥80	P value^a^	P for trend^b^
						
	N = 40 836	N = 7209 (17.6%)	N = 9312 (22.8%)	N = 10 683 (26.2%)	N = 6587 (16.1%)	N = 4962 (12.2%)	N = 2083 (5.1%)		
Age (year), median (IQR)	53.5 (43.5, 64.7)	33.5 (29.6, 36.8)	45.3 (42.7, 47.7)	54.8 (52.5, 57.2)	64.5 (62.1, 67.2)	74.6 (72.2, 77.1)	83.3 (81.5, 86.0)	<0.001	<0.001
**Clinical division**									
Health examination and division of family medicine	18 845 (48.9)	5427 (89.4)	6411 (74.4)	4595 (44.8)	1737 (26.6)	566 (11.4)	109 (5.2)	<0.001	
Urology division	14 836 (38.5)	395 (6.5)	1749 (20.3)	4699 (45.9)	3713 (56.8)	3104 (62.6)	1176 (56.5)		
Other division	4840 (12.6)	252 (4.2)	458 (5.3)	954 (9.3)	1091 (16.7)	1287 (26.0)	798 (38.3)		
**Number of PSA measurement during follow-up**									
Median (IQR)	1 (1, 3)	1 (1, 3)	1 (1, 3)	1 (1, 2)	1 (1, 2)	1 (1, 2)	1 (1, 2)	<0.001	<0.001
≥3 times, n (%)	10 398 (25.5)	1907 (26.5)	2668 (28.7)	2624 (24.6)	1646 (25.0)	1202 (24.2)	351 (16.9)	<0.001	<0.001
**Comorbidity, n (%)**									
Diabetes	2232 (6.2)	24 (0.5)	152 (2.0)	572 (5.8)	608 (9.5)	630 (12.8)	246 (11.9)	<0.001	<0.001
Hypertension	3108 (8.6)	41 (0.8)	208 (2.7)	695 (7.1)	754 (11.7)	925 (18.8)	485 (23.4)	<0.001	<0.001
Cardiovascular disease	4052 (11.3)	72 (1.4)	269 (3.5)	803 (8.2)	959 (14.9)	1257 (25.5)	692 (33.4)	<0.001	<0.001
CKD stage									
1–2	31 397 (87.3)	6925 (99.6)	8470 (98.4)	8474 (92.6)	4219 (78.9)	2437 (60.0)	872 (47.5)	<0.001	<0.001
3–5	4560 (12.7)	27 (0.4)	139 (1.6)	679 (7.4)	1130 (21.1)	1622 (40.0)	963 (52.5)		
**Baseline medication, n (%)**									
5-alpha reductase inhibitors	152 (0.5)	2 (0.1)	1 (0.0)	11 (0.1)	37 (0.7)	64 (1.4)	37 (1.9)	<0.001	<0.001
Finasteride	76 (0.3)	1 (0.0)	0 (0.0)	2 (0.0)	20 (0.4)	38 (0.8)	15 (0.8)	<0.001	<0.001
Dutasteride	77 (0.3)	1 (0.0)	1 (0.0)	9 (0.1)	17 (0.3)	27 (0.6)	22 (1.1)	<0.001	<0.001
Alpha-blocker	9438 (33.2)	111 (3.5)	782 (15.0)	2485 (31.4)	2394 (43.0)	2485 (54.5)	1181 (59.7)	<0.001	<0.001
Tamsulosin	8914 (31.3)	105 (3.3)	735 (14.1)	2370 (30.0)	2256 (40.5)	2331 (51.1)	1117 (56.5)	<0.001	<0.001
Muscarinic receptor antagonist									
Tolterodine	350 (1.2)	6 (0.2)	39 (0.8)	79 (1.0)	74 (1.3)	101 (2.2)	51 (2.6)	<0.001	<0.001
Solifenacin	151 (0.5)	5 (0.2)	14 (0.3)	32 (0.4)	31 (0.6)	54 (1.2)	15 (0.8)	<0.001	<0.001
Oxybutynin	1608 (5.7)	11 (0.3)	114 (2.2)	335 (4.2)	415 (7.5)	512 (11.2)	221 (11.2)	<0.001	<0.001
Flavoxate	8 (0.0)	0 (0.0)	0 (0.0)	0 (0.0)	4 (0.1)	2 (0.0)	2 (0.1)	0.028	0.004
**Medication after first PSA measurement, n (%)**									
BPH^c^	1555 (4.3)	8 (0.2)	65 (0.9)	317 (3.3)	483 (7.6)	479 (9.8)	203 (9.9)	<0.001	<0.001
Finasteride	311 (0.9)	5 (0.1)	1 (0.0)	56 (0.6)	87 (1.4)	112 (2.3)	50 (2.4)	<0.001	<0.001
Dutasteride	1336 (3.7)	3 (0.1)	64 (0.8)	282 (2.9)	426 (6.7)	396 (8.1)	165 (8.0)	<0.001	<0.001
**Baseline serum, median (IQR)**									
PSA (ng/mL)	0.92 (0.56, 1.73)	0.69 (0.48, 1.02)	0.74 (0.50, 1.11)	0.90 (0.55, 1.54)	1.37 (0.72, 2.93)	1.90 (0.86, 4.23)	2.26 (0.97, 5.20)	<0.001	<0.001
PSA >4 ng/mL	4149 (10.2)	119 (1.7)	184 (2.0)	694 (6.5)	1141 (17.3)	1330 (26.8)	681 (32.7)	<0.001	<0.001
Serum creatinine (mg/dL)	1.00 (0.89, 1.10)	0.96 (0.88, 1.05)	0.96 (0.87, 1.05)	0.98 (0.87, 1.10)	1.00 (0.90, 1.20)	1.10 (0.92, 1.39)	1.14 (0.95, 1.45)	<0.001	<0.001
eGFR (mL/min/1.73m^2^)	88.3 (73.4, 100.3)	103.9 (93.2, 113.1)	95.2 (84.8, 104.6)	86.9 (75.6, 97.5)	77.5 (62.8, 89.9)	65.5 (50.0, 80.6)	58.5 (43.7, 73.0)	<0.001	<0.001
ALT (IU/L)	26 (19, 38)	28 (20, 42)	28 (21, 40)	26 (20, 37)	24 (19, 34)	22 (16, 31)	19 (15, 28)	<0.001	<0.001
**Baseline urine, median (IQR)**									
WBC (uL)	3 (2, 6)	3 (2, 6)	3 (1, 6)	3 (2, 6)	4 (2, 9)	6 (3, 15)	6 (3, 28)	<0.001	<0.001
RBC (uL)	3 (2, 8)	3 (1, 6)	3 (2, 6)	3 (2, 8)	4 (2, 11)	6 (3, 17)	6 (3, 38)	<0.001	<0.001

***Abbreviations*:** ALT, alanine aminotransferase; BPH, benign prostatic hyperplasia; CKD, chronic kidney disease; eGFR, estimated glomerular filtration rate; IQR, interquartile range; PSA, prostate-specific antigen; RBC, red blood cells; WBC, white blood cells.

a *P* values are calculated using Kruskal–Wallis test for continuous variables and chi-square test for categorical variables.

b *P* values for trend are calculated using Spearman’s correlation for continuous variables and Cochran–Armitage trend test for binary variables.

c BPH medications that often affect the PSA level.
