# Supplementary figures and images for: Age-specific percentile-based prostate-specific antigen cutoff values predict the risk of prostate cancer: A single hospital observation
Source: Biomedicine (Taipei). 2023 Sep 1;13(3):9–24. doi: 10.37796/2211-8039.1415 (PMC10627214; doi:10.37796/2211-8039.1415)

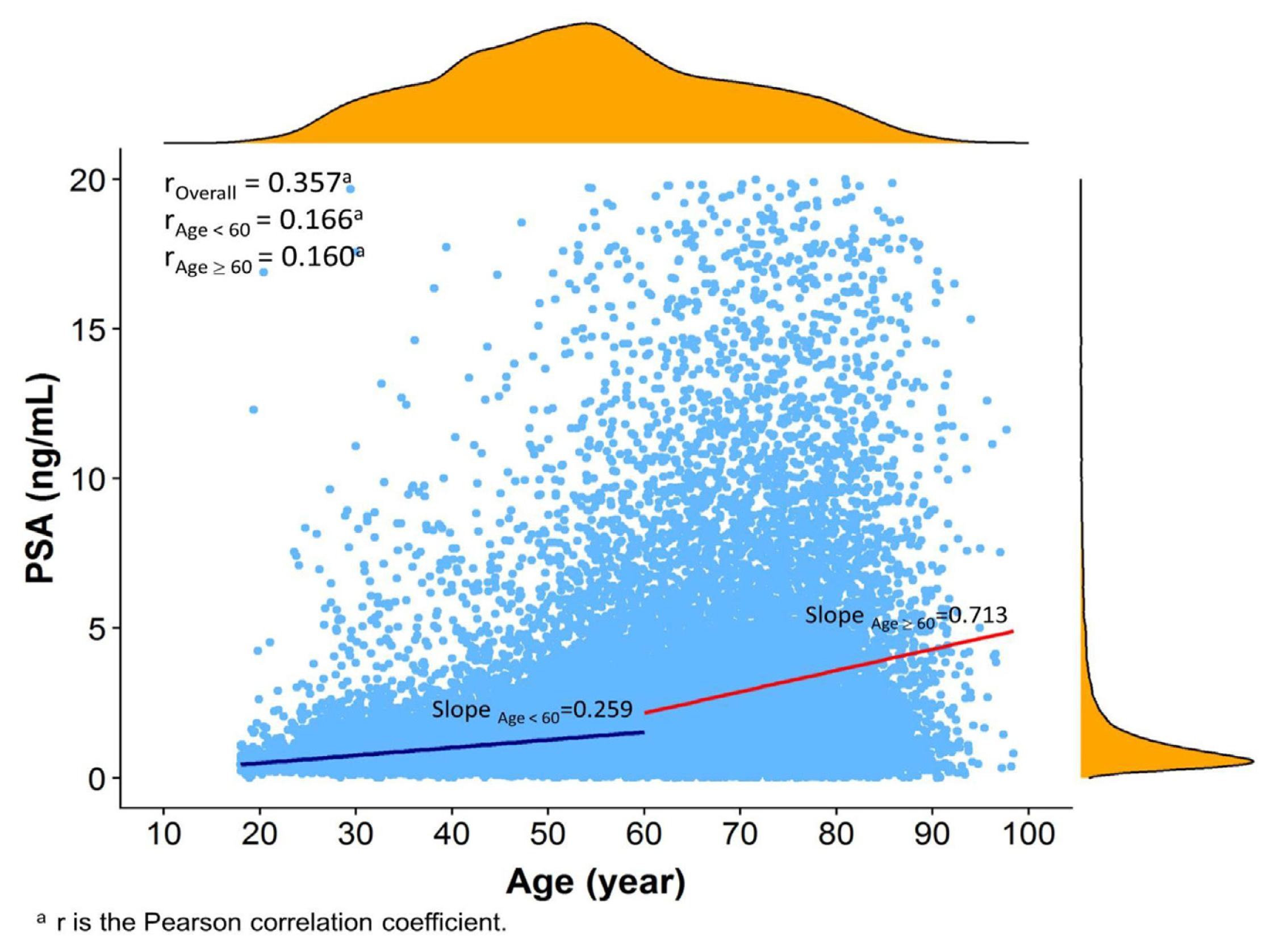

Supplement: Fig. S1 — Scatter plot of age at first prostate-specific antigen (PSA) measurement and PSA level among patients without subsequent prostate cancer. Abbreviations: PSA, prostate-specific antigen. [file bmed-13-03-009s1.tif]

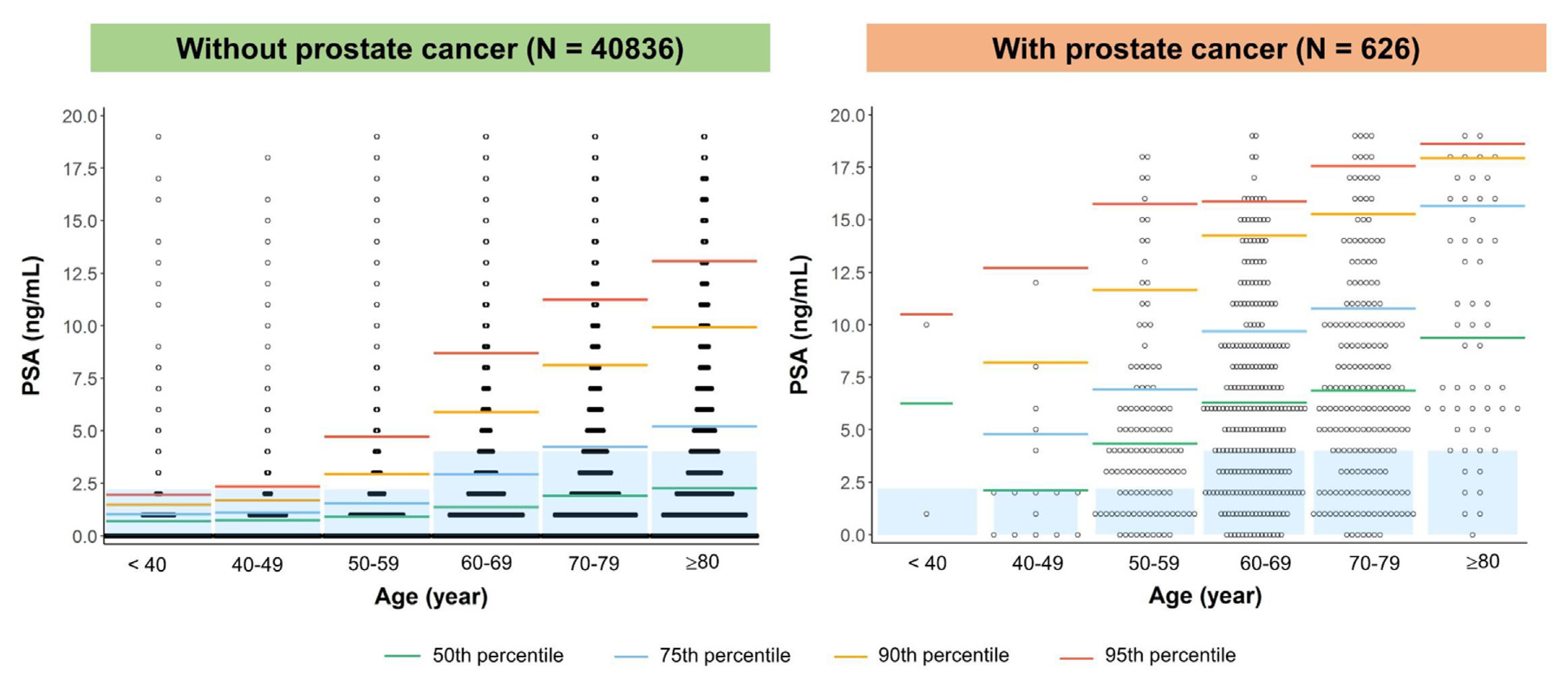

Supplement: Fig. S2 — Distribution plot of prostate-specific antigen (PSA) between patients with prostate cancer after first PSA measurement and those without prostate cancer. Shaded area indicates the PSA cutoff of 4 ng/mL for patients aged ≥60 years and 2.19 ng/mL for patients aged <60 years. Abbreviations: PSA, prostate-specific antigen. [file bmed-13-03-009s2.tif]
